# Red deer *Cervus elaphus* blink more in larger groups

**DOI:** 10.1002/ece3.9908

**Published:** 2023-03-15

**Authors:** Zeke W. Rowe, Joseph H. Robins, Sean A. Rands

**Affiliations:** ^1^ School of Biological Sciences University of Bristol Bristol UK; ^2^ Department of Ecological Sciences Vrije Universiteit Amsterdam Amsterdam Netherlands

**Keywords:** anti‐predator behaviour, elk, foraging behaviour, vigilance, visual ecology

## Abstract

Most animals need to spend time being vigilant for predators, at the expense of other activities such as foraging. Group‐living animals can benefit from the shared vigilance effort of other group members, with individuals reducing personal vigilance effort as group size increases. Behaviors like active scanning or head lifting are usually used to quantify vigilance but may not be accurate measures of this. We suggest that measuring an animal's blinking rate gives a meaningful measure of vigilance: increased blinking implies reduced vigilance, as the animal cannot detect predators when its eyes are closed. We describe an observational study of a captive population of red deer, where we measured the blinking rates of individual deer from groups of differing sizes (where mean group size ranged between 1 and 42.7 individuals). We demonstrate that as group size increases in red deer, individuals increase their blink rate, confirming the prediction that vigilance should decrease. Blinking is a simple non‐invasive measure and offers a useful metric for assessing the welfare of animals experiencing an increase in perceived predation risk or other stressors.

## Graphical abstract

Eye‐closure during blinking means that an animal is momentarily unable to collect information about its environment, and may expose it to predation. We show that the blink rate of red deer is positively correlated with group size, suggesting that blinking rate may indicate the vigilance level of individuals.

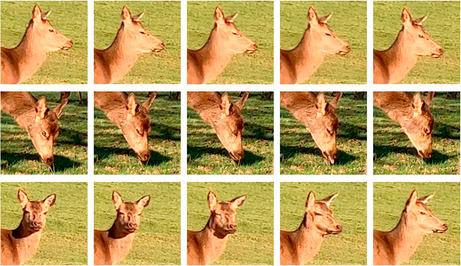

## 1 INTRODUCTION

Most animal species spend some part of their lives aggregated together in groups, and many benefits have been proposed and tested for this behavior (Krause & Ruxton, 2002; Ward & Webster, 2016). For prey species, grouping behavior can offer protection from predators through both the dilution of individual risk if an attack occurs (Bednekoff & Lima, 1998; Hamilton, 1971; Pulliam, 1973) and an increase in the chance of successfully detecting an approaching predator due to the combined vigilance effort of the group (Lima, 1990; McNamara & Houston, 1992; Pulliam, 1973), along with other anti‐predator advantages of grouping behavior such as synchronizing activity to dilute risk (Carere et al., 2009; Mónus & Barta, 2016; Rands, 2011; Rands et al., 2003; Rands & Ioannou, 2023). If an animal is being actively vigilant, it may be unable to conduct (or less efficient at) other important behaviors (like foraging or resting) at the same time (e.g. Fernández‐Juricic et al., 2005). Group membership means that vigilance can be pooled among the group members, which could mean that each individual can spend less time being vigilant and more time conducting other fitness‐enhancing behaviors. A rich body of theory and research has explored how group size and individual vigilance effort are related (Beauchamp, 2003, 2008, 2019; Beauchamp et al., 2021; Elgar, 1989), with much of it focussing on the prediction that individual vigilance effort will decrease as the group becomes larger. This prediction requires each individual to show a trade‐off between vigilance and other behaviors, where being actively vigilant either cannot occur at the same time as other behaviors or leads to a reduction in the efficiency of other behaviors that are conducted at the same time as being vigilant.

Anti‐predator vigilance is usually assumed to be occurring when an animal is actively scanning its surrounding environment with its head upwards, although there is no obvious consensus in how vigilance is defined in any particular species (see Allan and Hill (2018) for discussion of this problem in studies on primates). Although scanning behavior is likely to stop an animal from actively collecting food, this head‐up activity may not completely interfere with simultaneously conducted behaviors, such as chewing or social interaction. If a behavior that is recorded as vigilance allows an individual to do other things at the same time, then we may be falsely assuming that this behavior incurs the time and attention costs that are associated with vigilance (Treves, 2000). Without careful experimentation, it is difficult to assess how much of an individual's attention is devoted to vigilance when we observe scanning or other forms of vigilance‐like behavior, which may add to the huge variation (e.g. Beauchamp, 2019) in whether a study demonstrates that individual vigilance is related to group size or not.

Although it is difficult to define exactly when an individual is being vigilant, we may instead be able to define when it is *not* able to be vigilant. Blinking (the temporary closure of both eyes, involving movements of the eyelids: Blount, 1927) is a good example of an activity where an individual is momentarily unable to visually scan the environment. It is an essential maintenance behavior to keep the eyes moist and clean (Ranti et al., 2020) and is conducted tens of times every minute in some species of diurnal mammals (Rands, 2021; Stevens & Livermore, 1978; Tada et al., 2013) and birds (Kirsten & Kirsten, 1983). Although a blink takes only a fraction of a second, the sum of this loss of visual information over multiple blinks could be substantial for the individual. In humans, spontaneous blinking is accompanied by attentional suppression, where the individual experiences a blackout in visual attention for the duration of the blink, meaning that there is no awareness of the temporary blindness and lack of visual information while the blinking is occurring (Riggs et al., 1981; Volkmann et al., 1980). Blinking suppresses activity in both the visual cortex and other areas of the brain that are associated with awareness of environmental change (Bristow et al., 2005). If we assume that other animals show similar attentional suppression, then they are essentially blind and unaware of changes in their visual environment during each blink, which in turn means that they cannot be visually vigilant for predators. Even if they remain vigilant for olfactory and auditory cues during a blink, the loss of visual information will reduce the efficiency and timing of an animal's response to an approaching predator. Given that individuals are blinking tens of times every minute, both the duration of the individual blinks and the proportion of time that these multiple blinks occupy will impact on the information the animal is receiving from the environment.

An individual's blink rate, therefore, presents a trade‐off between the physiological benefits of blinking and the loss of visual information during the blink (Ranti et al., 2020). If an animal needs to dedicate more time to vigilance in a risky environment, then it has to reduce or suppress blinking to accommodate this increased vigilance. This is anecdotally demonstrated in American crows *Corvus brachyrhynchos*, which reduce their blink rates when looking at potentially dangerous stimuli (Cross et al., 2013), in horses *Equus caballus*, which decrease their spontaneous blink rate in response to stress‐inducing stimuli (Merkies et al., 2019), and in grackles *Quiscalus mexicanus*, which inhibit their blinking when observing human faces in varying orientations (Yorzinski et al., 2021). This link between blink rate and vigilance implies that blink rate will also be related to group size. As group size increases, theory predicts that individual vigilance can be reduced (Pulliam, 1973) and so any requirement to suppress blinking will be relaxed. Blink rate may, therefore, increase with an increase in group size. Evidence supporting this is still sparse: a comparison of chickens *Gallus gallus* feeding solitarily or in pairs showed an increase in blink rate and proportion of time spent blinking in the group‐feeding birds (Beauchamp, 2017), while a comparison of the blink rates of olive baboons *Papio anubis* (Matsumoto‐Oda et al., 2018) showed individuals in a small group blinked less than those in a large group (although the two groups were studied in different years). Here, we test this hypothesis by observing the blink rates in a captive herd of group‐living red deer *Cervus elaphus*. Red deer are well‐studied ungulates that exist both in wild populations and in managed captivity (Blaxter et al., 1974; Clutton‐Brock et al., 1982; Lovari et al., 2018) and are a sister species to wapiti (or North American elk, *C*. *canadensis*), where considerable work has been conducted exploring how vigilance behavior is mediated by natural predator presence (e.g. Childress & Lung, 2003; Creel et al., 2009; Halofsky & Ripple, 2008; Laundré et al., 2001; Liley & Creel, 2008; Lung & Childress, 2007; Winnie & Creel, 2007; Wolff & Van Horn, 2003). Given that vigilance has been shown in wapiti to be related to the sex and age of an individual (Childress & Lung, 2003; Wolff & Van Horn, 2003), we included these individual characteristics in our analysis.

## 2 METHODS

### 2.1 Study area, time, and subjects

This observational study was conducted on the herd of red deer within the 40.5 hectare deer park in Ashton Court Estate, Bristol, England (51.4440°N, 2.6378°W), which is composed mainly of open grassland, with scattered forestry and a small area of water. The herd, managed by Bristol City Council, consists of *c*. 110 individuals of varying age and sex, who appear to mix freely. The enclosure is open to the public outside of the rutting season, so the deer are habituated to both dogs, humans, motor vehicles, and occasional horses, and may be able to hear the vocalizations of a nearby (but separately fenced) captive herd of fallow deer *Dama dama* (Hoyle et al., 2021). Our observations were conducted over five days during the rutting season; observations were restricted between 12:00 a.m. and 4:30 p.m., so they were outside of the dawn and dusk peaks of regular rutting activity (Clutton‐Brock et al., 1982).

Ethical permission to conduct the study was given by the University of Bristol Animal Welfare and Experimental Review Board (#UB/16/062), and fully complied with both UK regulations and the ASAB/ABS guidelines for animal behavior research.

### 2.2 Behavioral sampling and observations

A random individual was selected as described in previous research on this herd (Rands et al., 2014). Briefly, the number of individuals present within the field of vision was visually estimated, and a random number generator was used to select an integer number within this count. A coin was tossed to choose whether counting started at the left‐most or right‐most individual in the field of vision, and then, moving across the field of vision, the deer were counted until the predetermined number was reached. The selected individual was observed and dichotomously aged (mature or young) and sexed (male or female). The individual was sexed by the presence of antlers, as after 1 year of age only males have antlers (Mitchell et al., 1977). Age was identified by an individual's size and morphology (larger individuals were older). If the individual was observed suckling, it was discarded, and randomization was repeated, as young individuals are hard to sex and exhibit behaviors uncommon to the rest of the herd (Clutton‐Brock et al., 1982). A count of the total number of young/mature males and females, along with suckling young, was made on three different days. The rounded averages of these five demographic classes were calculated and used in the pseudoreplication analyses presented.

**FIGURE 1:**
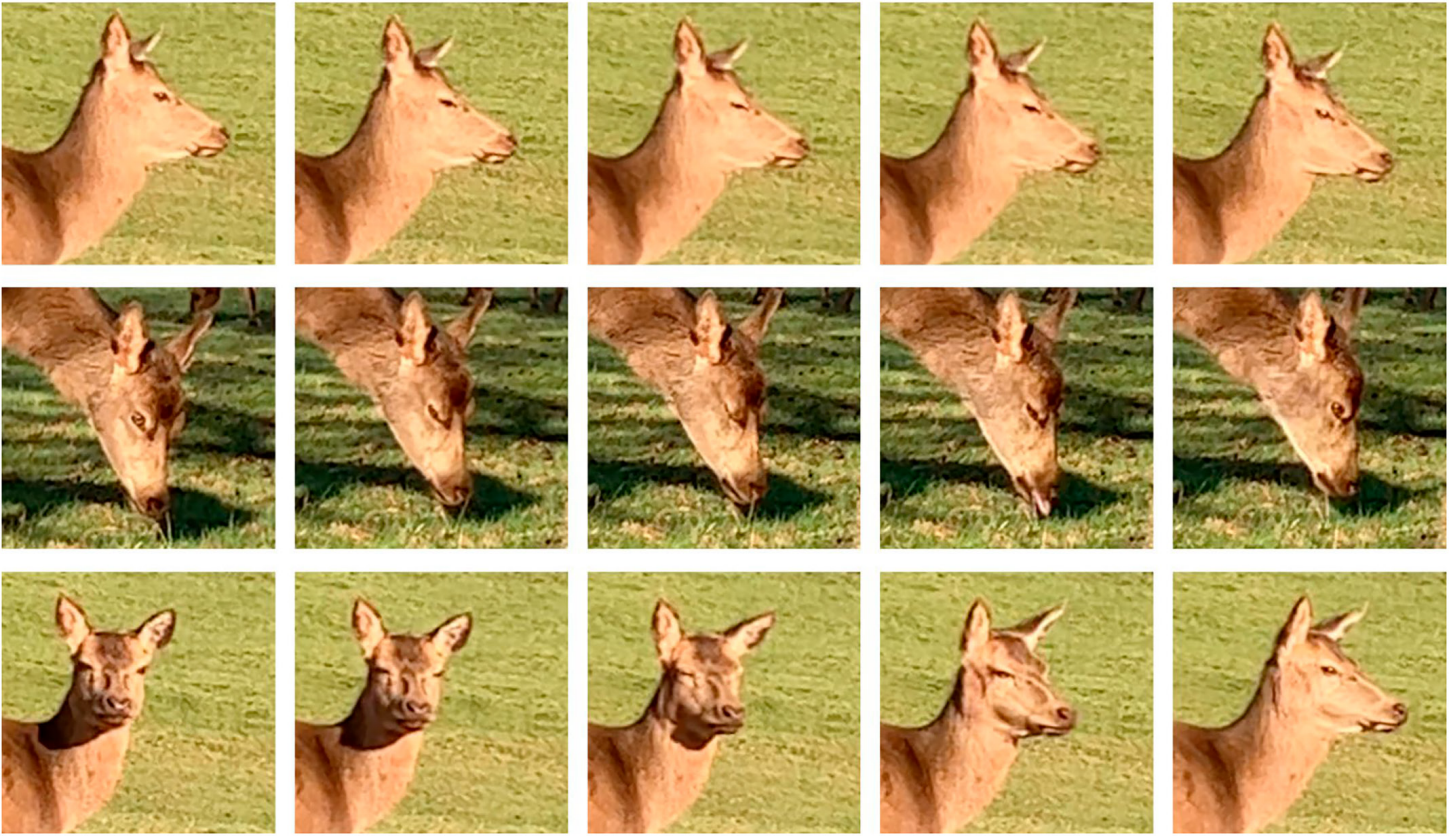
Three examples of blinking deer, showing blinks for different head positions. Each series of five panels shows a full blink and represent (from left to right) the eyes fully open immediately before the blink occurs, semi‐closure, full closure, semi‐opening, and fully open immediately after the blink. Note that the panels are given for the visual illustration of a blink and are not equally spaced in time.

Prior to the study, the observers (ZWR and JHR, who both conducted the measurements described) were trained in identifying the recorded behaviors, and pilot trials where both observers simultaneously and independently scored behaviors ensured repeatability of measurements. A blink was defined as a rapid full closure of the eye as sketched in Figure 1 and could occur regardless of head position. Group size was arbitrarily defined as the number of individuals aggregated no more than five body lengths away from at least one member of the group containing the focal deer, meaning a measured group was composed of individuals associated by a chain rule of association (see Rands (2015) for discussion of defining groups by associated neighbors within arbitrary distances). Before starting any set of observations, the observers waited 10 min at the site to habituate the deer. Observations were conducted approximately 10–100 m away from focal individuals using a 30× zoom spotting scope (Avian ED82 Magnesium Scope). An observation for a single selected individual was recorded for a maximum of 10 min. At the start of each minute, the group size was counted by one observer, with blink rate (blinks per minute) being continuously recorded for each minute by the other observer. If the deer's eye(s) were obstructed or there was a human/animal disturbance the observations were stopped with the current minute's measures being disregarded. Because the observers were unable to move easily during observations, this means that data collected was biased toward observable herds that were either stationary (either sitting or standing) or else moving slowly. A total of 75 observations of randomly selected individuals were observed using this method (and the analysis below describes how we controlled for potential pseudoreplication due to repeated samples potentially being taken using the same individual).

### 2.3 Statistical analysis

Data were recorded as blinks per minute with group size recorded for that minute. Individuals were recorded a mean of 4.9 (±2.0 SD) minutes before the data collection had to be discontinued due to the observer's view of the eyes being obstructed. For each individual, we calculated the mean number of blinks in a minute, and the mean group size per minute (where mean group size per minute ranged from 1 to 42.74 group members). To compensate for any unevenness caused by some mean values being based on more observations than others, we conducted the same analyses using just the first minute of data for all individuals (see the annotated *R* code for details): these data gave qualitatively similar results to the analysis involving mean group sizes and are not discussed further.

Using *R* 4.0.3 (R Development Core Team, 2019), we constructed a linear mixed effects model where the natural logarithm of mean blink rate was described by the natural logarithm of group size, and the maturity and sex of the focal individual, including the observation date as a random effect. Logarithms were used to satisfy model assumptions of normalized residuals. A full model including interactions was initially considered, but no interaction terms were significant and so the basic additive model with the three explanatory variables was used.

Although attempts were made to avoid replicating observations on the same individual during an observational day, it is likely that the 75 datapoints include some repeat observations of the same individual, given that the herd had 97 observable individuals (of which there were estimated to be 11 mature males, 23 young males, 26 young females and 37 mature females; 9 nursing young were also observed, but not included in the analysis), and individuals within each class could not be individually identified accurately. To explore potential effects of this pseudoreplication, we conducted simulations where identities were randomly allocated to individuals in each age class, and all but one datapoint for each ‘assumed individual’ was removed from the dataset. A linear model was then run on this filtered subsample of the dataset, harvesting the significance value. By conducting 100,000 independent repeats of this resampling, we could identify how likely our dataset was to give a significant result (assuming significance was set at *p* = .05), if we had only sampled each individual once.

## 3 RESULTS

**FIGURE 2:**
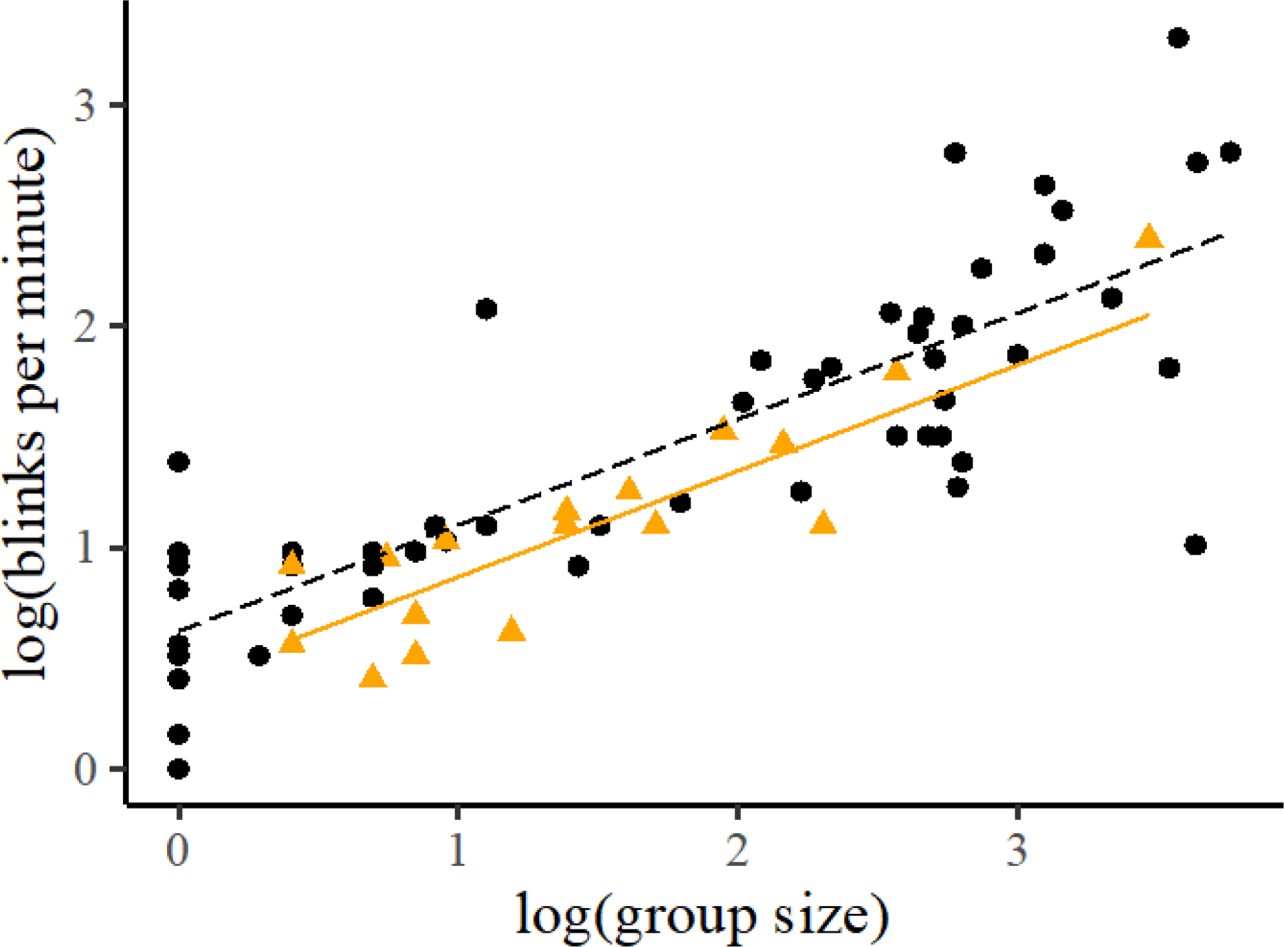
Scatterplot showing that blink rate increases with group size and maturity (where orange triangles and the fitted solid line represent young individuals, and black circles and the fitted dashed line represent adults).

Blink rate increased with group size (*t*_67_ = 11.38, *p* < .001; Figure 2), and adults blinked more than young deer (*t*_67_ = 2.11, *p* = .038; Figure 2). There was no relationship between sex and blinking (*t*_67_ = 0.35, *p* = .727). Considering potential pseudoreplication of unidentifiable individuals, resampling showed that group size remained significant (at *p* < .001) in 100% of simulations. There was a significant (at *p* < .05) effect of sex in only 0.17% of simulations, while maturity was significant in 40.73% of simulations. So, the effect of maturity observed could potentially have been an effect of resampling the same individuals, but the increasing blink rate in response to increasing group size is unlikely to have been affected by any pseudoreplication caused by repeated sampling of the same individual.

## 4 DISCUSSION

Our results demonstrate that blinking increases as group size increases. Given that blinking interferes with vigilance behavior, and that individual vigilance is predicted to decrease as groups get larger, this supports our argument that the blink rate represents a trade‐off between gaining visual information through vigilance and the physiological benefits of blinking. We note that these results are only correlational, and we suggest that a link between vigilance and blinking could be demonstrated with suitable experimental manipulation of perceived risk (e.g. Abbey‐Lee et al., 2016; Fey et al., 2010; Mónus & Barta, 2016; Rands & Cuthill, 2001).

We argued earlier that observed behaviors that are typically recorded as vigilance (such as holding a head upright or active scanning – see Beauchamp, 2015 and Mónus, 2018 for discussion) may not be conducted solely for vigilance (assuming that we are only considering visual vigilance, acknowledging that prey species will also be relying on auditory and olfactory information which will not be interrupted by eye closure). Similarly, blinking may not solely be a maintenance behavior that is traded‐off against being able to collect visual information. Blinking may include a social element, as rhesus macaques, *Macaca mulatta*, are able entrain their blink rate in response to social cues, coordinating their blinking with partners that they were interacting with (Ballesta et al., 2016). Our results suggest that the proportion of scanning time that an individual red deer allocates to vigilance is related to the size of its immediate group, but we should acknowledge that scanning behavior may be influenced by social behavior as well as vigilance. Studies of wild wapiti showed that young individuals were less vigilant than older ones (Childress & Lung, 2003; Lung & Childress, 2007), and suggest that this may be because the younger individuals are unlikely to be able to outrun a predator if one appears. If the young in our study are following this behavioral pattern, this also suggests that blinking may not be completely correlated with vigilance level, as the younger deer should have had higher blink rates when compared to mature adults if the young were being less vigilant. Young deer lack experience of potential threats, may have different nutritional requirements and schedules to adults, and may not move their heads in a similar manner to adults, all of which could cause a difference between the age classes. This result may also reflect some form of social signaling between adults, although we note that we did not see a difference between males and females (which contradicts results from wapiti suggesting that vigilance patterns may be sex‐ and age‐determined (Childress & Lung, 2003; Lung & Childress, 2007)) – we note here that we conducted our observations during the rutting season where males were competing for access to females, and this result either indicates that vigilance behavior is not being directed towards potential sexually driven intraspecific conflict, or that it is similar between the two sexes for potentially different reasons. Other aspects of social behavior may also be important for determining blink rates, such as position within the group (echoing the vigilance changes seen in wapiti, where individuals on the outside of a group tend to be more vigilant; Lung & Childress, 2007; Winnie & Creel, 2007). Theory predicts that individuals on the outside of the group should be more vigilant than those in the middle, and anecdotal evidence from olive baboons suggests that peripheral individuals may blink less (Matsumoto‐Oda et al., 2018). It may be possible to test many of the standard predictions connecting vigilance and group size (e.g. Beauchamp, 2003, 2008, 2019; Elgar, 1989) using blink rate as a proxy for vigilance.

Observational studies on free‐ranging wild wapiti living in national parks with varying levels of natural predation have variously found no effect of group size on vigilance (measured as heads‐up scanning behavior) in males and females (Halofsky & Ripple, 2008; Laundré et al., 2001; Lung & Childress, 2007; Winnie & Creel, 2007), females alone (Wolff & Van Horn, 2003), and one study (Childress & Lung, 2003) saw no significant relationship in males or breeding females and a decrease in vigilance with respect to increasing group size in non‐breeding females. Model comparison (Liley & Creel, 2008) suggests that wapiti vigilance may show a non‐linear relationship with herd size, where vigilance increases with small numbers, and then decreases after the herd has reached an intermediate size, while position in the herd was less important. These results suggest that wapiti do not normally show a decrease in individual vigilance in response to increasing group size, as would be predicted by standard theory (e.g. Beauchamp, 2003, 2008, 2019; Elgar, 1989), which in turn either suggests that this predicted relationship does not occur, or that standard measures of vigilance using assays of head‐up scanning behavior may not be suitable for quantifying vigilance, as we have argued earlier. We did not assay heads‐up scanning behavior alongside the blinking behavior measured here, and a sensible next step would be to measure both simultaneously, to assess whether red deer conform with wild wapiti in showing a similar lack of correlation between head‐up scanning vigilance and group size.

It could be argued that our semi‐captive deer population is not suitable for assaying something which is considered an anti‐predator behavior, as the population lives in a relatively benign managed environment, with no natural predators present. In wild wapiti, the very real risk of predation by wolves (which have been recently reintroduced) or other carnivores has a measurable impact on behavior, affecting both observable behavior and use of space in the environment (Halofsky & Ripple, 2008). Wapiti may respond to patchiness of predation risk in the environment by choosing where they spend their time, which in turn can mediate whether their diet changes (Hernández & Laundré, 2005), and whether they show a stress response (e.g. Creel et al., 2009).

However, recent arguments suggest that a human‐dominated environment may have significant impact on the landscape of fear experienced by animals (Moleón & Sánchez‐Zapata, 2023; Palmer et al., 2022, 2023). There is evidence to suggest that red deer living in what we consider to be a predator‐free environment do nonetheless show responses to anthropogenic cues and features in the environment that they may be treating in the same way as a predator cue. Free‐ranging wapiti were shown to increase vigilance and their likelihood of flight behavior in response to vehicle presence (Forrest & St Clair, 2009; Preisler et al., 2006), and vigilance behavior has been observed as being more influenced by both traffic and transport‐related infrastructure than by predator presence (Ciuti et al., 2012). Other evidence suggests that red deer mediate their behavior greatly in response to human presence. Vigilance was more likely in areas with human disturbance in the Scottish Highlands (Jayakody et al., 2008). Wild free‐ranging individuals in southern Germany were shown to avoid areas with high human recreational presence during the day (Coppes et al., 2017), and a comparison of free‐ranging populations from different regions of Poland with differing natural predation levels showed that fecal glucocorticoid concentrations were lowest and least variable in high‐predation areas, and that concentrations indicating high stress were instead likely to be linked to the level of anthropogenic disturbance that they were experiencing (Zbyryt et al., 2018). Similarly, measurement of fecal glucocorticoids in two herds of semi‐wild red deer living in parkland in England similar to the current study demonstrated that assayed stress was higher on days with higher visitor numbers (Dixon et al., 2021). All this evidence suggests that red deer confined to a small area with constant disturbance by both pedestrian visitors and motor vehicles may well show stress responses and anti‐predator behavior that may be differently expressed when compared to deer living in an undisturbed wild environment with natural predators present. It would be interesting to examine whether these different stressors cause different responses, to disentangle whether the blinking response that we see is coupled with ‘head‐up’ scanning vigilance, or whether scanning is not related to group size as is seen in the multiple wild wapiti studies described above. This could be done by considering red deer in environments where wolves are present, or else by manipulating ‘natural’ predator cues such as by adding wolf urine to the environment (Apfelbach et al., 2005), although red deer quickly acclimatize to this manipulation (Elmeros et al., 2011). However, it is also useful to question whether the lack of ‘natural’ predators is going to stop the deer performing the vigilance behaviors that they have evolved. As a relevant example, we could consider Père David's deer *Elaphurus davidianus*; this endangered species has only existed in a managed, predator‐free environment for over a thousand years but, nonetheless, shows distinct group size‐related vigilance behavior in response to human disturbance (Zheng et al., 2013), demonstrating that vigilance behavior does not need wolves or tigers to be visible in a species.

Blink rate may also be influenced by factors other than the size of the group, including rainfall and wind (which have been shown to influence blink rate in captive grackles; Yorzinski, 2020a; Yorzinski & Argubright, 2019). Similarly, the behavior that an individual conducts simultaneously to the blink may be important. Experiments in humans suggest a mechanism controlling the timing of blinks, which occurs to minimize the chance of missing crucial information (Maffei & Angrilli, 2019; Nakano et al., 2009; Ranti et al., 2020; Shin et al., 2015; Wiseman & Nakano, 2016), with evidence of similar behaviors in rhesus macaques *Macaca mulatta* (Ballesta et al., 2016). Peafowl *Pavo cristatus* also time their blinks to coincide with gaze shifts (Yorzinski, 2016) while grackles blink less during flight behaviors (Yorzinski, 2020b) and chickens blink more when feeding when compared to scanning (Beauchamp, 2017), all minimizing the time where visual information cannot be collected from the environment. Therefore, if an individual is moving, the timing and frequency of its blinks may reflect this movement. Anecdotally, blinking does not necessarily have to occur at the same time as movement (and vice versa) in red deer (S. Rands, *pers*. *obs*.), but it would be enlightening to measure how correlated blinking is with head movement to explore whether there is attentional control. It would also be sensible to assay blinking in response to group size in resting deer groups, which would not be undergoing head and body movements that could confound the measure of blinking that is recorded. Similarly, animals in different attentional states may change their frequency of blinking, such as during sleep in herring gulls *Larus argentatus* (Amlaner & McFarland, 1981).

Our results suggest that the measurement of blinking presents a simple and non‐invasive technique for observing attention that can be conducted remotely. Although we conducted our sampling in the field, this could be done using video footage. Being able to analyze video footage means that information about blink duration can also be collected, and previous studies have demonstrated that this additional metric can also vary between individuals and species (Beauchamp, 2017; Matsumoto‐Oda et al., 2018; Rands, 2021; Tada et al., 2013; Yorzinski, 2020a; Yorzinski & Argubright, 2019), and may increase in relation to group size (Beauchamp, 2017). From a field observation perspective, being able to zoom in on detail may also be extremely useful if the observed individuals are a long distance away (as we acknowledge we were lucky with being able to get within 100 m of our sample animals). Given that blinking has been shown to decrease under stressful conditions (Cross et al., 2013; Merkies et al., 2019), this simple technique could help us to understand the welfare requirements of managed animals that normally live in social groups.

## AUTHOR CONTRIBUTIONS

**Zeke Rowe:** Conceptualization (equal); investigation (equal); methodology (equal); writing – review and editing (equal). **Joe Robins:** Conceptualization (equal); investigation (equal); methodology (equal); writing – review and editing (equal). **Sean Rands:** Conceptualization (equal); data curation (equal); formal analysis (equal); investigation (equal); methodology (equal); supervision (equal); visualization (equal); writing – original draft (equal); writing – review and editing (equal).

## CONFLICT OF INTEREST STATEMENT

The authors declare no competing interests.

## Data Availability

The dataset and annotated *R* code are freely available on *figshare* at https://doi.org/10.6084/m9.figshare.17920322.
